# Elements of Electro-Metallurgy

**Published:** 1843-04

**Authors:** 


Art. IX.—*Elements of Electro-Metallurgy.* By Alfred Smee, f.r.s., Surgeon to the Bank of England, &c. &c. Second Edition, revised, corrected, considerably enlarged, illustrated with Electrotypes and numerous Woodcuts.—*London,* 1843. 8vo, pp. 338.

We certainly live in such an age of scientific novelties as none have ever witnessed before. And these novelties are not of abstract or speculative interest alone; but are brought into practical employment, supplying desiderata, of which scarcely any but the dreamy speculator, or the ingenious romancer, ever contemplated the advent. There is scarcely one of the famous Century of Inventions soberly put forth by the Marquess of Worcester two centuries ago, or of the speculations on which the philosophers of Laputa were represented, by the biting satirist of the last age, as absurdly engaged, which is not now realized. The locomotive engine, and the electric telegraph almost annihilate time and space in terrestrial communication. We are promised a means of aerial communication (which, as we are gravely assured, only waits the completion of certain legal operations, to be carried into effect on an extensive scale) fully as certain and speedy,—touching which we maintain for the present a philosophic incredulity. We have learned the art of transforming the fleeting shadow into the permanent lights and shades of the most perfect drawing. And by the art, which Mr. Smee has done much to advance, and of the present state of which the work before us gives a full and highly interesting account, the most perfect and durable facsimile may be obtained of any highly-wrought surface, without the least injury to the original. It is a remarkable instance of the value of an *idea,* that the deposition of metallic copper, in that form of the voltaic battery, known as Professor Daniell's, was known for some time to take place, in such a manner as to produce a complete counterpart, even to the minutest scratch, of the surface on which it takes place, before there was any thought of applying it to practical purposes. The idea occurred, we believe, about the same time to Professor Jacobi, of St. Petersburg, and to Mr. Spencer, of Liverpool. Once suggested, it has been laid hold of and applied to a great variety of purposes, of which Mr. Smee's work contains a full account. To such of our readers as are desirous of keeping up that general information, which often contributes so much to the estimation and usefulness of a practitioner, we recommend the perusal of this treatise; and we need scarcely add, that those who can find time to amuse themselves with the operations it describes, will find in its pages ample guidance. The example of Mr. Smee himself shows that the zealous pursuit of such an object is not inconsistent with professional success.

